# The complete plastome of *Ctenolophon englerianus* Mildbr. (Ctenolophonaceae)

**DOI:** 10.1080/23802359.2019.1673684

**Published:** 2019-10-07

**Authors:** Zi-Xun Wang, Dong-Min Jin, Guo-Dong Wang, Ting-Shuang Yi

**Affiliations:** a Key Laboratory of Ministry of Education for Medicinal Plant Resource and Natural Pharmaceutical Chemistry, National Engineering Laboratory for Resource Developing of Endangered Chinese Crude Drugs in Northwest of China, College of Life Sciences, Shaanxi Normal University, Xi'an, China;; b Germplasm Bank of Wild Species, Kunming Institute of Botany, Chinese Academy of Sciences, Kunming, China;; c College of Life Sciences, University of Chinese Academy of Sciences, Beijing, China

**Keywords:** *Ctenolophon englerianus*, Ctenolophonaceae, plastome, phylogenomics

## Abstract

*Ctenolophon englerianus* Mildbr. is endemic to West Africa. The wood of this species is very strong, and is widely used as building material in local regions. In this study, we determined its complete plastome sequence. This is the first reported complete plastome sequence in the family Ctenolophonaceae. The plastome of *C. englerianus* was found to possess a total length of 161,553 bp containing two inverted repeats (IRs) of 27,469 bp, a large single copy (LSC) region of 89,386 bp, and a small single copy (SSC) region of 17,229 bp. The plastome contains 110 unique genes, consisting of 76 protein-coding genes, 30 tRNA genes and 4 rRNA genes. The *rpl32, rps16* and *infA* genes were lost. To validate the phylogenetic relationships of *C. englerianus* in Malpighiales, we have selected seven representative families from three major clades of Malpighiales to construct phylogenetic tree. According to the phylogenetic topologies, *C. englerianus* has a close relationship with *Erythroxylum novogranatence*.

*Ctenolophon englerianus* is one of two species of the only genus in the family Ctenolophonaceae (Van Hooren et al. 1988). Both species of *Ctenolophon* are trees that may be recognised by their opposite, entire leaves with interpetiolar stipules (Van Hooren et al. 1988; Angiosperm Phylogeny Group 2016). Their grey-drying and closely-ribbed fruit is distinctive. *Ctenolophon englerianus* is only distributed in West Africa (Kubitzki 2014). The wood of *C. englerianus*, known as ‘okip’ in Gabon, is widely used as house building materials, poles, or railway sleepers (Badré 1973). The newly sequenced plastome will be a helpful information for the genomic and genetic studies of this species. At the same time, the reported plastome is useful for further evolutionary and phylogenetic studies in Malpighiales.

The plant of a *Ctenolophon englerianus* was sampled from Ogooue-Maritime, Gabon (02°30′S 009°44′E). Voucher specimen (Gordon McPherson-16911) was deposited in the herbarium of Missouri Botanical Garden. In this study, we determined the complete plastome of *C. englerianus* based on the whole-genome Illumina sequencing dataset. The experiment procedure is as reported in Jin et al. (2019). We used the DNeasy Plant Mini Kit to extract total DNA, then performed fragmentation according to the manufacturer's manual (Illumina) to construct short insert (212 bp) library. Paired-end (PE) sequencing was performed on Illumina HiSeq X TEN at Plant Germplasm and Genomics Center (Kunming Institute of Botany). The paired-end reads were filtered and assembled into a complete plastome using GetOrganelle v1.6.2a (Camacho et al. 2009; Bankevich et al. 2012; Langmead and Salzberg 2012; Jin et al. 2018), with final assembly graph checked in Bandage (Wick et al. 2015). The plastome was automatically annotated using PGA (Qu et al. 2019) and GeSeq (Tillich M et al. 2017), then manually adjusted in Geneious 8.0.2 (Kearse et al. 2012). The final complete plastome was deposited in GenBank (accession number MN313429). The plastome of *C. englerianus* is 161,553 bp in length, containing two inverted repeats (IRs) of 27,469 bp, a large single copy (LSC) region of 89,386 bp, and a small single copy (SSC) region of 17,229 bp. The plastome contains 110 unique genes, consisting of 76 protein-coding genes, 30 tRNA genes and 4 rRNA genes. The *rpl32*, *rps16* and *infA* genes were lost. The overall GC content in the plastome of *C. englerianus* is 36%, for which the corresponding value of the LSC, SSC, and IR region are 33.5%, 29.9%, and 42.1%, respectively.

In order to verify the phylogenetic relationship of *C. englerianus* in Malpighiales, we constructed the maximum likelihood (ML) tree of seven families of the Malpighiales that have plastomes, the *Averrhoa carambola* L. of Oxalidales and *Vitis rotundifolia* Michx. of Vitales have been used as outgroup species (Figure 1). A phylogenetic analysis of a dataset including 82 gene sequences was performed using RAxML version 8.1.21 with 1000 bootstrap replicates (Stamatakis 2014). Consistent with previous results (Xi et al. 2012), Ctenolophonaceae was strongly supported as the sister of the Erythroxylaceae – Rhizophoraceae clade. The genome data in this paper can be subsequently used for genomic and genetics studies of this species, and phylogenetic studies in Malpighiales.

**Figure 1.**
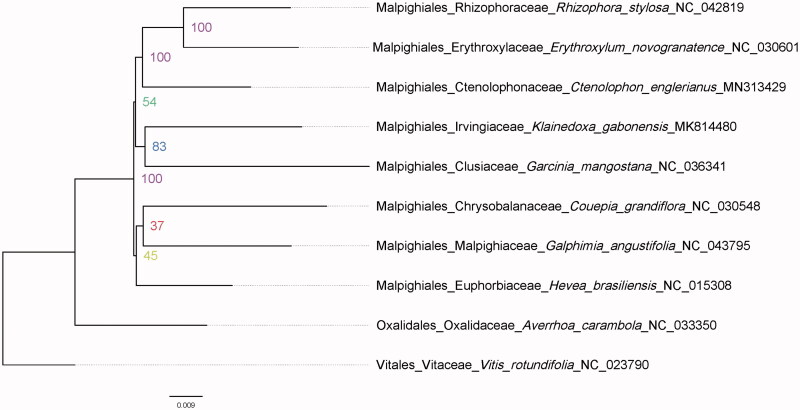
The ML phylogenetic tree of *Ctenolophon englerianus* with other 7 families of Malpighiales based on the 82-gene (78 protein-coding and 4 rRNA genes) matrix. The number at each node indicate the ML bootstrap values. *Averrhoa carambola* and the *Vitis rotundifoliaare* were selected as outgroup.
